# Supplementary material for: Perinatal suicidal behavior in sub-Saharan Africa: A study protocol for a systematic review with meta-analysis
Source: PLoS One. 2023 May 8;18(5):e0285406. doi: 10.1371/journal.pone.0285406 (PMC10166536; doi:10.1371/journal.pone.0285406)
Supplement: S1 Appendix — (DOCX) [file pone.0285406.s002.docx]

**Appendix A. Literature Search Strategy**

| **Table A1. Database: PubMed** |
| --- |
| 1. "prenatal"[All Fields] OR "prenatally"[All Fields] OR "prenatals"[All Fields] OR "antepartum"[All Fields] OR ("antenatal"[All Fields] OR "antenatally"[All Fields]) OR ("postpartum period"[MeSH Terms] OR ("postpartum"[All Fields] AND "period"[All Fields]) OR "postpartum period"[All Fields] OR "postpartum"[All Fields]) 2. **''suicide ideation OR suicidal intent OR suicidal gesture OR suicidal attempt* OR suicidal behavior*''[MeSH Terms]** 3. **''sub saharan Africa OR east Africa OR west africa OR central africa Or northern africa''[MeSH Terms]** 4. 1 AND 2 AND 3 |
| **Table A2. Database: Embase**   1. perinatal/exp OR perinatal*:ti,ab OR antepartum*:ti,ab OR antenatal*:ti,ab OR postpartum*:ti,ab OR postnatal*:ti,ab 2. suicide/de OR suicide*:ti,ab OR suicidal ideation*:ti,ab OR suicidal intent*:ti,ab OR suicidal gesture*:ti,ab OR suicidal attempt*:ti,ab OR suicidal behaviour/exp OR behav*:ti,ab 3. ‘‘sub sahran africa’’/de OR ‘‘sub saharan’’*:ti,ab OR ‘‘east africa’’*:ti,ab OR ‘‘**west africa’’***:ti,ab **OR ‘‘central africa’’***:ti,ab **Or ‘‘northern africa’’***:ti,ab 4. 1 AND 2 AND 3 |
| **Table A3. Database: PsycINFO**   1. Prenatal OR antenatal OR antepartum OR postnatal OR postpartum 2. **‘‘suicide ideation’’ OR ‘‘suicidal intent’’ OR ‘‘suicidal gesture’’ OR ‘‘suicidal attempt’’* OR ‘‘suicidal behavior’’*** 3. **‘‘sub saharan Africa’’ OR ‘‘east Africa’’ OR ‘‘west africa’’ OR ‘‘central Africa’’ Or ‘‘northern africa’’**   4 1 AND 2 AND 3 |
