# Supplementary material for: Perinatal suicidal behavior in sub-Saharan Africa: A study protocol for a systematic review with meta-analysis
Source: PLoS One. 2023 May 8;18(5):e0285406. doi: 10.1371/journal.pone.0285406 (PMC10166536; doi:10.1371/journal.pone.0285406)
Supplement: Attachment — Submitted filename: PLOS ONE_SUBMISSION_REVIEW PONE-D-22-22410.docx [file pone.0285406.s003.docx]

In my opinion, PONE-D22-22410 is a very interesting work, the topic is relevant not only for the African reasearch, but also for the perinatal mental health research in the world. However, the authors must consider the following. After a preliminary review of the submission, following the publication criteria of PLOS ONE, based on the AMSTAR and PRISMA checklists systematic reviews, it is my assessment:

According to AMSTAR guidelines, the following items are missing from the article:

1. Research question(s)
2. Objective (s) of the systematic review
3. Inclusion criteria must include: population, intervention, and comparator group (PICO)
4. Explanation of the selection of the study designs (If the authors will include RCTs, NRSI or both).

Based on PRISMA CHECKLIST (S1 Table), the following items are missing from the article:

1. **Administrative information:** identification and registration (must be on page 3 instead of 4).
2. **Introduction:** Objectives are missing and also the explicit statement of the question(s) the review will address according to PICO.
3. **Methods:** in the eligibility criteria are missing the study characteristics: PICO, study design, time frame and report characteristics. And also are missing the items such as: Outcomes and priorization, Meta-bias, and Confidence in cumulative evidence.

These resuIts of the preliminary review have led me to believe it is necessary to have the author work on the missing items mentioned above to complete their protocol before any reviewers can be invited to provide their assessment.

Best regards,

María Eugenia Gómez López, PhD

Academic Editor
