# Supplementary material for: Perinatal suicidal behavior in sub-Saharan Africa: A study protocol for a systematic review with meta-analysis
Source: PLoS One. 2023 May 8;18(5):e0285406. doi: 10.1371/journal.pone.0285406 (PMC10166536; doi:10.1371/journal.pone.0285406)
Supplement: Attachment — Submitted filename: Response to Reviewer.docx [file pone.0285406.s004.docx]

**Manuscript title: Perinatal suicidal behavior in Sub-Saharan Africa: A study protocol for a systematic review with meta-analysis**

**Manuscript ID No: PONE-D22-22410**

Dear esteemed Editor, We are so grateful for your quick and positive response to our article. We appreciate your considerate and intellectual comments and suggestions. We addressed each of the comments and suggestions point-by-point as mentioned hereunder.

In my opinion, PONE-D22-22410 is a very interesting work, the topic is relevant not only for the African research, but also for the perinatal mental health research in the world. However, the authors must consider the following. After a preliminary review of the submission, following the publication criteria of PLOS ONE, based on the AMSTAR and PRISMA checklists systematic reviews, it is my assessment:

According to AMSTAR guidelines, the following items are missing from the article:

1. **Research question(s)**

**Authors’ response:** Dear editor, thank you so much for your intellectual suggestions. Accordingly we included the research question in the revised version of the manuscript.

2. **Objective (s) of the systematic review**

**Authors’ response:** Thanks in advance. We have included the objective of the study in the last section of the introduction of the revised manuscript.

3. **Inclusion criteria must include: population, intervention, and comparator group (PICO)**

**Authors’ response:** Dear Editor! Thanks a lot for your insightful suggestion. We included the PICO search guideline under the eligibility criteria. Thanks once again.

4. **Explanation of the selection of the study designs (If the authors will include RCTs, NRSI or both).**

**Authors’ response:** Dear editor, we detailed mentioned the types studies to be included (in terms of study design) in the eligibility criteria. In the review we will consider all quantitative observational studies (cross-sectional, case-control, cohort, or longitudinal studies, and survey findings) and randomized control trial (RCT) studies will be included.

**Based on PRISMA CHECKLIST (S1 Table), the following items are missing from the article:**

1. **Administrative information: identification and registration (must be on page 3 instead of 4).**

**Authors’ response:** Thank you so much for the recommendation. We have edited accordingly.

2. **Introduction: Objectives are missing and also the explicit statement of the question(s) the review will address according to PICO.**

**Authors’ response:** Thank you for your constructive comments. We have incorporated the comments in the main document.

3. **Methods: in the eligibility criteria are missing the study characteristics: PICO, study design, time frame and report characteristics. And also are missing the items such as: Outcomes and priorization, Meta-bias, and Confidence in cumulative evidence.**

**Authors’ response:** Dear editor, thank you for your constructive comments. We have incorporated all the comments in the main document.

Journal Requirements:

1. Please ensure that your manuscript meets PLOS ONE's style requirements, including those for file naming.

**Authors’ response:** We ensured that our manuscript meets PLOS ONE's style requirements.

2. PLOS ONE does not copy edit accepted manuscripts (https://journals.plos.org/plosone/s/criteria-for-publication#loc-5). To that effect, please ensure that your submission is free of typos and grammatical errors.

**Authors’ response:** We have checked and then corrected all typos and grammatical errors

3. We note that you have stated that you will provide repository information for your data at acceptance. Should your manuscript be accepted for publication, we will hold it until you provide the relevant accession numbers or DOIs necessary to access your data. If you wish to make changes to your Data Availability statement, please describe these changes in your cover letter and we will update your Data Availability statement to reflect the information you provide.

**Authors’ response:** Thanks. Since our article is a study protocol, we stated the Data Availability as “The identified research data will be made publicly available when the study is completed and published”, as per the PLOS ONE’s Data Availability statement for protocol. We updated the Data Availability in the cover letter.

4. We note that this manuscript is a systematic review or meta-analysis; our author guidelines therefore require that you use PRISMA guidance to help improve reporting quality of this type of study. Please upload copies of the completed PRISMA checklist as Supporting Information with a file name “PRISMA checklist”.

**Authors’ response:** Thanks a lot for your suggestion. We have uploaded the PRISMA checklist.
