# Supplementary material for: Perinatal suicidal behavior in sub-Saharan Africa: A study protocol for a systematic review with meta-analysis
Source: PLoS One. 2023 May 8;18(5):e0285406. doi: 10.1371/journal.pone.0285406 (PMC10166536; doi:10.1371/journal.pone.0285406)
Supplement: Attachment — Submitted filename: Response to Reviewers.docx [file pone.0285406.s005.docx]

**Response to Reviewers Comments**

**Manuscript ID:** PONE-D-22-22410R1

**Title:** Perinatal suicidal behavior in Sub-Saharan Africa: A study protocol for a systematic review with meta-analysis
**Dear Editor and Reviewers:** Thank you so much for your time with our manuscript and your constructive comments and suggestions, which greatly helped in revising our protocol. We accepted all the comments and suggestions and amended the manuscript accordingly. The point-by-point responses to the reviewers’ comments are listed below.

**Reviewer 1**

1) Does suicidal behavior include suicidal ideation? right now this point is unclear, although the authors included suicidal ideation in the search strategy and defined suicidal ideation, it is not covered in the definition of suicidal behaviors defined in Definition of concepts.

**Authors’ response**: Dear Editor, thank you so much for your intellectual suggestions. Frankly speaking, suicidal behavior does not include suicidal ideation. We thought this could be explained by some screening instruments used only for research purposes. However, for the current review, we planned to state that it was suicidal behavior. We will accept reviewer comments to modify and include suicidal ideation in the suicidal behavior as follows: ‘‘Suicidal behavior- includes suicidal ideation, intent, thought attempt, and completion." So we have presented a reference to an earlier review article as evidence. (Kessler and Ustun, 2004).

2) For eligibility criteria, why exclude studies published before the year 2000?

**Authors’ response**: Thank you so much for your comments. Actually, we don’t have a scientific basis for this issue. Authors have been interested in observing the trend over the past 20 years, with the rough assumption that most of the studies conducted on suicidal behavior are at least 20 years old. However, we have accepted and excluded time limitations. Thanks again.

3) The description of the search strategy is somewhat hard to follow. Can the authors list the main search strategy with the key terms and then report the variations used for each key term? or if the authors have other better ways to present the search strategy it will be acceptable as long as easy to follow.

**Authors’ response:** Thank you so much for your comments. We have addressed the comment and uploaded the database search strategy as supplementary information. See supplementary information 1.

**Reviewer 2**

**Abstract:**
In the context of maternal morbidity, the ICD-10 classifies suicide as a direct cause of death, not an indirect cause of death as stated here. Please review same.
**Authors’ response:** Thank you so much for your intellectual suggestion. We have amended the mentioned section as per your information.

"Perinatal mental illnesses" are mental illnesses that exist during the perinatal period so this statement is misleading as it conveys a sense that they are separate, when they are not. Please revise for clarity.

**Authors’ response:** Thanks for your suggestion. We have revised the mentioned section.

The perinatal period alone does not increase susceptibility to suicidal behaviour. Again, this is misleading and should be reviewed.

**Authors’ response:** Thanks a lot for your suggestion. We have revised the suggested sentence.
For the abstract, there is too much detail provided in the methods section and a synopsis only should be provided.

**Authors’ response:** Thanks. We have revised the mentioned section accordingly.

**Introduction:**
Again perinatal mental illnesses are mental illnesses that exist during the perinatal period so this statement is misleading as it conveys a sense that they are separate, when they are not. Please revise for clarity.

**Authors’ response:** Thanks for your suggestion. We have revised the mentioned section.
Suicide is a symptom and not a condition. Please revise for clarity.
**Authors’ response:** Thanks a lot. We have revised the mentioned word.
"Suicidal behaviour WAS thought to be a leading..." if this is past-tense, what is currently thought?
**Authors’ response:** Thanks a lot. We have revised the mentioned tense.
Again, the perinatal period alone does not increase susceptibility to suicidal behaviour. This is nuanced and should be reviewed.

**Authors’ response:** Thanks a lot. We have revised the mentioned section.
Again, in the context of maternal morbidity, the ICD-10 classifies suicide as a direct cause of death, not an indirect cause of death as stated here. Please review same.
**Authors’ response:** Thanks you so much. We have revised the mentioned information accordingly.
"During the perinatal period women have..." - this is not reflective of all countries and is too nuanced to be this clear-cut. There is evidence that despite women having regular contact with HCP's, women's mental health needs are not addressed and therefore, this needs to be reviewed to reflect this.

**Authors’ response:** Thank you so much your intellectual suggestions. We have revised the mentioned paragraph.
**Specific objectives**
Please define what is meant by 'level'
**Authors’ response:** Thanks a lot. It was to mean “prevalence”. We have replaced it with “prevalence” in the revised version.
**Specific questions:**
Please define what is meant by 'determines it'
**Authors’ response:** Thanks for your suggestion. It was to mean “what determines that prevalence”
**Methods:**
Please provide rationale for choosing 21 year timeframe.

**Authors’ response:** Thanks for your intellectual suggestion. Actually, we have no any solid rationale for choosing 21 year timeframe for our review rather the researchers (our) interest. We have unlimited the publication year depending on your suggestion. Thanks a lot.
Psych Info - please amend to read PsycINFO

**Authors’ response:** Thanks a lot. We have amended the mentioned section accordingly.
Please consider language in search terms; "committed" is no longer an accepted term in the context of suicide.

**Authors’ response:** Thank you so much for your intelligent suggestion. We will not consider "committed" in the search terms.
**Screening procedure:**
Please consider an alternative phrase to "get rid of" (omit, remove etc.)

**Authors’ response:** Thank you so much. We replaced the phrase "get rid of" with “omit” as per your comment. Thanks

**Discussion**
It is accepted little evidence exists in terms of sub-Saharan Africa, however, several reviews have been conducted on the prevalence rates and psychosocial risk factors relating to perinatal suicide/suicidal ideation or suicidal behaviours - please provide commentary on the state of the evidence currently and what gaps this review aims to address.

**Authors’ response:** Dear Editor! Thanks a lot for your constructive suggestions.

Suicide remains the leading cause of direct maternal death in the first postnatal year (Saving Lives, Improving Mothers’ Care 2021 report), and it is among the top three causes of pregnancy-associated deaths, as defined by the CDC (Lindahl, V., Pearson, J. L., & Colpe, L. (2005). Some studies have also shown black women are twice as likely as white women to report having suicidal thoughts (suicidal ideation) in the immediate postpartum period (doi: 10.1089/jwh.2015.5346). In terms of causes of adolescent deaths, suicide ranked third globally and first in Africa (11.2 per 100 000 people) (WHO, 2021).

Considering an earlier review on suicidal ideation and behavior in low and middle-income countries, two (2) studies have been done so far, and the differential explanation is presented below. A systematic review conducted by Daniela C. Fuhr et al. (2014) focused on the identification of the studies reporting the proportion of pregnancy-related deaths from injuries and suicide in low- and middle-income countries. So this study focused only on completed types of suicide and injuries from related psychosocial risk factors, in contrast to the current study, which focuses on all types of suicidal forms, which include suicidal ideation, intent, gesture, and attempt, as well as suicide deaths. Secondly, the a review by Lindahl V, Pearson JL, and Colpe L. (2005) was done worldwide, but only single studies in South Africa were included from Africa. As stated earlier, it is quite different compared to the current one in terms of study setting (continent: worldwide vs. sub-Saharan), the timing of suicide (pregnancy and postpartum vs. antepartum, pregnancy, and postpartum), and timing (included study before 2005 E.C.). Furthermore, the Joan M et al. (2022) study excluded suicide and focused on the perinatal mental health of pregnant teenagers in sub-Saharan Africa. The results of this study (Joan M et al., 2012) and the other reviews discussed above may have shed some light on the concept. However, it is improbable that a clear correlation between suicidal ideation and behavior and a significant psychosocial factor affecting overall health has been identified in the region.

So the assessment of suicide in the context of maternal health, in particular during the perinatal period, in sub-Saharan African countries needs to be considered a public health priority, and there is no doubt that any mental health policies could not be efficiently implemented without adequate evidence of the situation on the ground. Therefore, this study will be imperative to collect and combine empirical data on suicide among mothers in the perinatal period, and doing so will help to provide better evidence to plan psycho-social interventions in the particular context of maternal mental health.

**Limitations**
Please ensure all language is future-tense, such as English language WILL be included as opposed to was included. Also I would query the line "in most studies, the death of the mother..." this reads as though the study has been completed so it is important not to confuse the protocol with the findings of the review.
**Authors’ response:** Thank you so much for your suggestion. We have corrected all the tense errors the mentioned section accordingly.
**General:**
There are issues with the reference list - please ensure to address these.

**Authors’ response:** Thanks a lot. We have checked and revised all the errors in the references list.
**Journal Requirements:**
Please review your reference list to ensure that it is complete and correct. If you have cited papers that have been retracted, please include the rationale for doing so in the manuscript text, or remove these references and replace them with relevant current references. Any changes to the reference list should be mentioned in the rebuttal letter that accompanies your revised manuscript. If you need to cite a retracted article, indicate the article’s retracted status in the References list and also include a citation and full reference for the retraction notice.

**Author response:** Thank you so much for your important suggestion. We have checked the reference list for its completeness and correctness. We have not cited any retracted articles. Moreover, we updated reference lists 8 and 22. Moreover, we have checked and corrected all errors in the references list.
